# Development of Alectinib-Suspended SNEDDS for Enhanced Solubility and Dissolution

**DOI:** 10.3390/pharmaceutics14081694

**Published:** 2022-08-14

**Authors:** Eun Ji Park, Seung Ah Choi, Kyoung Ah Min, Jun-Pil Jee, Sung Giu Jin, Kwan Hyung Cho

**Affiliations:** 1 College of Pharmacy and Inje Institute of Pharmaceutical Sciences and Research, Inje University, Gimhae 50834, Korea; 2 College of Pharmacy, Chosun University, 309 Pilmun-daero, Dong-gu, Gwangju 61452, Korea; 3 Department of Pharmaceutical Engineering, Dankook University, 119 Dandae-ro, Dongnam-gu, Cheonan 31116, Korea

**Keywords:** suspended self-nanoemulsifying drug delivery system, alectinib HCl, dissolution, ultrasonication

## Abstract

Alectinib hydrochloride (ALH), a tyrosine kinase inhibitor, is a practically water-insoluble drug classified as BCS class IV. The present study aimed to develop novel suspended self-nanoemulsifying drug delivery system (Su-SNEDDS) to enhance the solubility and dissolution rate. The Su-SNEDDS was prepared by saturation and suspension of ALH in SNEDDS with ultrasonication energy. According to evaluation by the dispersion test and the results of particle size analysis, the selected SNEDDS composed of Kolliphor HS 15 and Capmul MCM C8 as surfactant and oil, respectively, showed a complete dissolution within 30 min. However, the SNEDDS loaded and solubilized only small amount of ALH (<0.6%, *w*/*w*). On the other hand, 10% ALH-loaded Su-SNEDDS containing small and micronized ALH particles of <5 μm had about 20-fold higher ALH-loading% than the SNEDDS and reached a 100% dissolution rate within 30 min in 1% sodium lauryl sulfate (SLS) pH 1.2 buffer. In the dispersion test and microscopic observation, micronized ALH particles in the Su-SNEDDS were readily dispersed in the dissolution medium with spontaneous nanoemulsion formation and instantly solubilized with the aid of SLS. Taken together, our results suggest that the Su-SNEDDS would be a potent oral dosage form to enhance the solubilization and dissolution rate of ALH in a new technological way.

## 1. Introduction

Alectinib hydrochloride (ALH), an anaplastic lymphoma receptor tyrosine kinase (ALK) inhibitor, is used for the treatment of ALK-positive locally advanced or metastatic non-small-cell lung cancer. ALH is not only more potent than the first-generation inhibitor crizotinib but also inhibits acquired ALK-resistant mutations [1]. ALH is known to inhibit and treat signal transduction and protein expression by abnormal ALK genes, such as mutations and tumor cell proliferation. The ALK gene mutation is one of the main causes of lung cancer and is found in 3–5% of lung adenocarcinomas [2].

**Figure 1:**
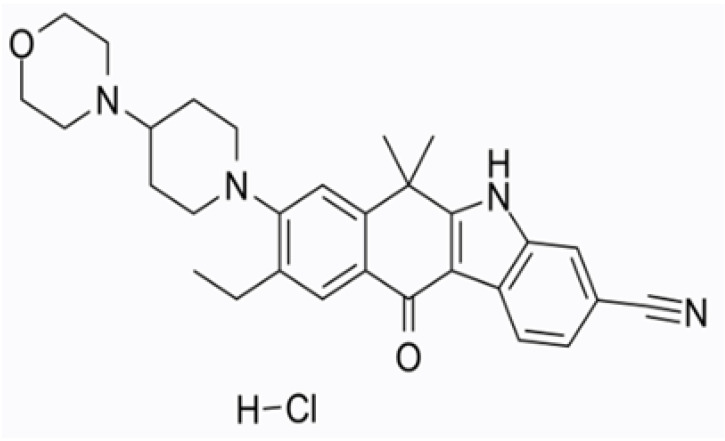
Structure of alectinib hydrochloride.

The structure of ALH comprises a benzo–carbazole group containing hydrocarbon rings (Figure 1)**,** which makes this molecule water-insoluble and fairly hydrophobic. ALH is classified as Biopharmaceutical Classification System (BCS) class IV and has low drug permeability (2.5 × 10^−4^ cm/s) as well as low solubility in the aqueous buffer (0.015 μg/mL at pH 7.90) and bioavailability of 37% (with food) [3,4,5]. Therefore, the physicochemical properties of ALH are very poor and a formulation to improve solubility and absorption in vivo should be secured.

Various solubility and dissolution enhancements previously used in the pharmaceutical area include self-nanoemulsifying drug delivery systems (SNEDDS), microparticles, inclusion complexes, microspheres, solid dispersion, and pH modification [6,7,8,9,10,11]. Among these technologies, the SNEDDS, a homogeneous liquid mixture of surfactant, oil, and drug, to form nanoemulsion when stirred in an aqueous medium, has been used for the formulation of poorly soluble drugs [12,13]. When the SNEDDS is diluted with aqueous medium by stirring, droplet size of 20–200 nm is spontaneously formed. This feature can improve drug solubility and absorption, thereby improving bioavailability regardless of food intake [14]. Although the SNEDDS has many advantages, drug loading may be limited if its solubility in oil is not sufficiently high. To solve this problem, supersaturated SNEDDS studies have been conducted; however, there remains the problem that large amounts of oils and surfactants should be used to achieve the targeted drug loading [15].

Suspended SNEDDS (Su-SNEDDS) is a suspended form of the supersaturated drug in the SNEDDS. It is a formulation designed to increase a targeted drug loading and the advantages of the SNEDDS. In general, supersaturated SNEDDS is thermodynamically unstable and drugs in supersaturated formulations may crystallize during storage or after oral administration [16]. Moreover, Ostwald ripening, a phenomenon of non-uniform structural change over time due to re-deposition from small particles with a relatively higher chemical potential to larger particles with a relatively lower chemical potential, may occur and instability may appear [17]. Therefore, when the Su-SNEDDS is formulated in a general way, unstable form and reduced dissolution may appear. The Su-SNEDDS preconcentrate prosed in this work is a formulation technology that combines suspension technology with the SNEDDS using ultrasonication. In the present study, we used this technique to increase drug loading, solubility, and the dissolution rate. Ultrasonication can obtain finely crushed uniform particles by projecting ultrasonic waves onto liquids and particles. This recently used nanoemulsion technology is being used in various formulations. The amount of energy transfer varies with temperature, sonication power, and time and has a significant effect on particle size. In particular, it can play a role in increasing solubility by transforming poorly water-soluble drugs into micronized and uniform particles [18].

In the present study, we aimed to develop a novel Su-SNEDDS to increase the drug loading, solubility, and dissolution of ALH, a poorly water-soluble drug. In order to develop the Su-SNEDDS, solubility evaluation to select suitable excipients, particle size evaluation according to the type and ratio of vehicle components, drug solubility, and particle size evaluation using ultrasonic suspension technology were performed. The Su-SNEDDS was formulated with excipients consisting of surfactants, oils, and drug, using ultrasonication suspension techniques. The solubility and dissolution evaluations of ALH-loaded SNEDDS and Su-SNEDDS were then performed. In addition, the physical and chemical properties were investigated in terms of particle size measurement using microscopy and a particle size analyzer and a scanning electron microscope (SEM).

## 2. Materials and Methods

### 2.1. Materials

Alectinib hydrochloride was purchased from Wuhan Vanz Pharm Inc. (Wuhan, China). Kolliphor HS 15 (polyoxyl 15 hydroxy stearate), Kollisolv PEG 300 (polyethylene glycol 300), Kollisolv PEG 400 (polyethylene glycol 400), Kolliphor EL (polyoxyl 35 castor oil), Kolliphor PS 20 (Polysorbate 20), Kolliphor PS 80 (polysorbate 80), Kolliphor RH 40 (polyoxyl 40 hydrogenated castor oil), and Kollisolv MCT 70 (medium-chain capric triglyceride) were kindly provided by BASF (Ludwigshafen, Germany). Transcutol P (diethylene glycol monoethyl ether) and Capryol 90 (propylene glycol monocaprylate (type II)) were kindly provided by Gattefosse (Saint-Priest, France). Capmul MCM C8 (mono/diglycerides of caprylic acid) and Capmul MCM C10 (mono/diglycerides of capric acid) were kindly provided by ABITEC Corporation (Janesville, WI, USA). Sodium lauryl sulfate (SLS) was kindly provided by DUKSAN Pure Chemical (Ansan, Korea). All other chemicals were of reagent grade and were thus used without further purification.

### 2.2. HPLC Condition

The HPLC analysis of samples was conducted using the Waters 2695 HPLC system (Waters, Milford, MA, USA) equipped with a UV-Vis detector (Waters 2487, Waters, Milford, MA, USA). ALH in samples was analyzed using a reverse-phase column with C18, 5.0 μm, 4.6 mm × 25 cm (Shiseido, Tokyo, Japan). The mobile phase consisted of methanol and phosphate buffer of pH 3.0 (70:30, *v*/*v*). The phosphate buffer of pH 3.0 was prepared with the adjustment of the mixture of 0.7 mL phosphoric acid and 2 L water by the 2 N NaOH solution. The HPLC analysis was performed with 1.0 mL/min. The injected volume of the sample was 10 μL, and UV detection was monitored at 265 nm [19]. Data acquisition and processing were performed using the Waters LC Solution software.

### 2.3. Solubility on the Aqueous pH and SLS Concentration

The pH solubility was tested in distilled water, pH 1.2 (HCl/NaCl) buffer, pH 4.0 acetate buffer, and pH 6.8 phosphate buffer and in four different concentrations of SLS solution (1%, 3%, 5%, and 10%). An excess amount of ALH powder (approximately 10 mg) was added to each test medium of 1 mL. After stirring with a magnetic bar at room temperature for 24 h, the mixture was centrifuged at 15,000 rpm for 10 min. Then, a supernatant of 0.5 g was obtained, and 0.3 g of the supernatant was diluted 5-fold with a diluent (water:methanol = 50:50, *v*/*v*), and the amount of ALH was analyzed using the HPLC conditions as described above. The ALH amount was measured three times.

### 2.4. Solubility in Various Excipients

The solubility of ALH in various excipients such as surfactants, oils, and solubilizers was determined. This test was performed at 50 °C and 70 °C rather than room temperature to obtain high solubilization. An excess of ALH powder (50 mg) was added to a vial containing 1 g of each excipient. Thereafter, the solution in vial was stirred in oil bath at the heated temperature of 50 °C or 70 °C for 24 h to make saturated; 0.5 g of supernatant was obtained after centrifugation at 15,000 rpm for 10 min, and 0.3 g of the supernatant was diluted 5-fold with a diluent (water:methanol = 50:50, *v*/*v*), and the amount of ALH was analyzed using the HPLC conditions as described above. The ALH amount was measured three times.

### 2.5. Characterization of Various Vehicle Compositions

In order to select specific oils and surfactants that would exhibit the optimum dispersed particle size, various vehicle compositions without the drug were prepared and tested. Surfactant and oil were weighed to 1 g in vial at various weight ratios (1:9–9:1), stirred, and mixed while heating at 50 °C. The samples were then taken out and equilibrated at room temperature for 3 h. The particle size of the samples was measured with a particle size analyzer as described in the below Section 2.6.

### 2.6. Particle Size Measurement

Each 100 mg vehicle or SNEDDS was diluted in a vial with 2.9 mL distilled water by vortexing for 1 min. The particle size was determined using a particle size analyzer (NanoBrook 90Plus, Brookhaven instruments Corporation, Holtsville, NY, USA) at the wavelength of 659 nm and the scattering angle of 90°. The temperature was set at 25 °C, and the number of measurements was set at 5 cycles. The measurements were repeated three times.

### 2.7. Preparation and Solubility Test of ALH-Loaded SNEDDS and Su-SNEDDS

Based on the vehicle composition tests, ALH-loaded SNEDDS and Su-SNEDDS with various formulations (see Table 2) were prepared. First, surfactant and oil mixture were prepared and uniformly mixed using a stirrer at the heated temperature of 50 °C. Then, SNEDDS and Su-SNEDDS formulations were prepared by each of the following methods.

#### 2.7.1. Agitation

For ALH-loaded SNEDDS formulations with agitation, excess ALH (100 mg) was added to the 5 g mixture of surfactant and oil. ALH-loaded SNEDDS from agitation was prepared by thoroughly mixing at 70 °C with stirring for 60 min. The mixture was centrifuged at 2000× *g* for 15 min, and the supernatant was obtained as ALH-loaded SNEDDS formulations with agitation, and the amount of ALH was analyzed using the HPLC conditions as described above.

#### 2.7.2. Agitation and Ultrasonication

For ALH-loaded SNEDDS formulations with agitation and ultrasonication, excess ALH (100 mg) was firstly added to each 5 g mixture of surfactant and oil at 70 °C with stirring for 60 min, and ultrasonication was performed under 40 °C temperature limit setting with the amplitude of 30% (equal to 150 Watts electric energy) for 8 min of operation by VCX-500 (Sonics & Materials, Newtown, CT, USA). Then, the mixture was centrifuged at 2000× *g* for 15 min, and the supernatant was obtained as ALH-loaded SNEDDS formulations with agitation and ultrasonication, and the amount of ALH was analyzed using the HPLC conditions as described above.

For ALH-loaded Su-SNEDDS formulations, predetermined ALH amount (25.8 mg/77.0 mg/237.4 mg/530.5 mg, respectively) was added to 5 g of the previously prepared 0.5% ALH-loaded SNEDDS to give final concentration% (1%/2%/5%/10%) and suspended, followed by stirring at 500 rpm for about 15 min. Thereafter, ultrasonication by VCX-500 was performed with the same conditions as those of the above ALH-loaded SNEDDS [20].

### 2.8. Dissolution Test

Dissolution test of ALH-loaded SNEDDS, Su-SNEDDS, and raw ALH was performed using a 708-DS dissolution tester (Agilent Technologies Inc., Santa Clara, CA, USA). Each ALH-loaded SNEDDS and Su-SNEDDS equivalent amount of 30 mg (for 0.5% ALH-loaded SNEDDS, and 1%, 2% ALH-loaded Su-SNEDDS) or 150 mg (for 5%, 10% ALH-loaded Su-SNEDDS in suspended form of drug) ALH was filled into a gelatin capsule shell and then placed into a dissolution tester with a sinker. The dissolution test was performed under conditions of 50 rpm paddle speed in 900 mL of the pH 1.2 buffer with or without 1% SLS at 37 ± 0.5 °C. The samples were aliquoted in 5 mL increments with a syringe and collected at predetermined time intervals (5, 10, 15, 30, 45, 60, 90, and 120 min). The aliquoted sample was diluted 2-fold with a diluent (water:methanol = 50:50, *v*/*v*) and filtered through a 0.45 μm syringe filter (DISMIC^®^-13HP, ADVANTEC^®^, Tokyo, Japan) [21]. The filtered sample was analyzed using the HPLC condition as described above.

### 2.9. Microscopic Observation

Microscopic method was used to observe the ALH particles in ALH-loaded Su-SNEDDS and their dispersion. The microscope was a ZEISS Axio Observer Z1 microscope (Carl Zeiss microscopy, Thornwood, NY, USA) equipped with an Axio Cam Erc 5s camera (Zeiss, Jena, Germany). Dispersion samples of 1%, 2%, 5%, and 10% ALH-Loaded Su-SNEDDS (F6) in pH 1.2 buffer and 1% SLS pH 1.2 buffer were prepared by diluting 10-fold. Simultaneously, raw ALH and 1%, 2%, 5%, and 10% ALH-loaded Su-SNEDDS samples were also used directly without any diluting. After dropping a sample (2–3 drops) onto a silane-coated slide glass (Muto Pure Chemicals Co., Ltd., Tokyo, Japan) and covering it with a cover glass (diameter 15 mm, Matsunami Glass Ind., Ltd., Osaka, Japan). The slide glass on the microscope stage was fixed and images were taken using the ZEN 200 (Zeiss) program while adjusting each of the magnifications of 5-, 10-, and 20-fold [22].

### 2.10. Scanning Electron Microscope (SEM)

Representative surface morphology and approximate particle size from raw ALH and the representative dispersion of 2% ALH-loaded Su-SNEDDS in the pH 1.2 buffer were analyzed using a field emission scanning electron microscope (FE-SEM; S-4300SE; Hitachi, Ltd., Tokyo, Japan). Each sample’s 10-fold dilution was dispensed dropwise onto a TEM grid (01813-F; Ted Pella, Inc., Redding, CA, USA) and dried for 24 h to remove water completely. The prepared sample was placed on a carbon tape and vacuum coated. All measurements were conducted at ambient temperature [23].

## 3. Results

### 3.1. Solubility on the Aqueous pH and SLS Concentration

ALH is a structurally basic compound having a piperidine group and available with the drug form of the hydrochloride salt, which provides insufficient solubility in water. Moreover, the commercial product is inconvenient to take with meals to improve absorption and requires a large drug dose (1200 mg/day) [24]. To solve this problem, previous studies used anionic surfactants such as SLS, which has problems such as gastrointestinal irritation and diarrhea, and a fundamental solution has not been reached yet [4,9]. In this study, the SNEDDS and Su-SNEDDS were newly developed to increase drug loading and solubility. First, the solubility of ALH according to the pH and SLS concentration was evaluated. The results revealed a very poor solubility of <40 μg/mL in all tested pHs and water (see Table 1). In the solution of SLS, the solubility was high and increased up to 754.31 μg/mL at 10% SLS, which means that SLS works as a solubilizer to form a micelle with ALH [25].

### 3.2. Solubility in Various Excipients

**Figure 2:**
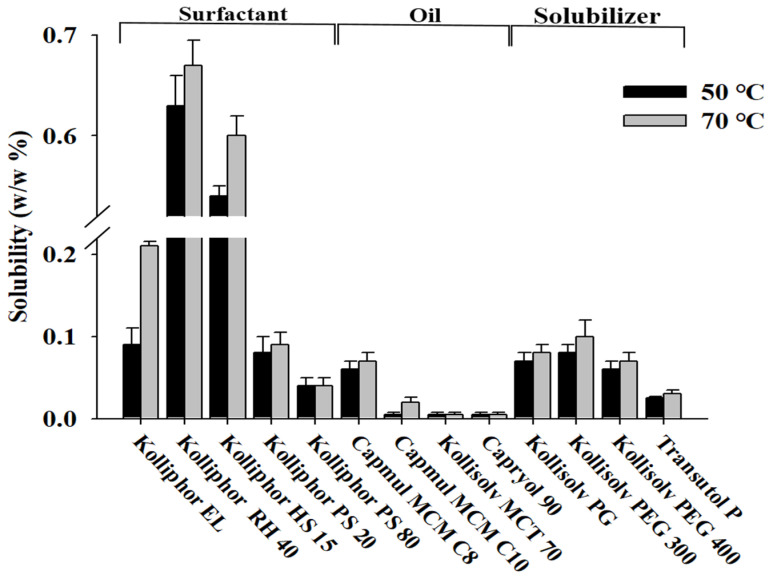
Solubility of ALH in various oils, surfactants, and solubilizers.

To select the optimal additive for the development of the Su-SNEDDS, the solubility of ALH was evaluated using various surfactants and oils (see Figure 2). Solubility evaluation was performed at 50 °C and 70 °C, where each additive became the liquid form in order to measure the solubility of highly viscous or semi-solid surfactants at room temperature. The solubility at 70 °C was overall higher than at 50 °C. At 70 °C, the surfactant (0.21–0.67%) was found to show about a 10-fold higher solubility than the oil (0.02–0.07%). Kolliphor RH 40 (0.67 ± 0.06%) and Kolliphor HS 15 (0.60 ± 0.08%) showed the highest solubility of ALH among the tested surfactants. In the case of the oil, the solubility was lower than that of the surfactant, but Capmul MCM C8 (0.07 ± 0.01%) and Capmul MCM C10 (0.02 ± 0.02%) showed good solubility. Despite the low solubility of ALH, these two oils were selected to compose the SNEDDS formulations. For the SNEDDS formulations, two surfactants and oils with higher solubility were selected. For the optimal formulation of the Su-SNEDDS, the additives that showed the highest drug solubility among surfactants and oils were selected [26]. The selected Kolliphor RH 40, Kolliphor HS 15, Capmul MCM C8, and Capmul MCM C10 are safe excipients applied to various systems to increase the bioavailability of poorly water-soluble drugs to enhance solubilization and absorption [27,28]. In addition, Kolliphor HS 15 is an excipient for parenteral use. Solubilizers were excluded due to their low solubility of less than 0.1%.

### 3.3. Characterization of Various Vehicle Compositions

**Figure 3:**
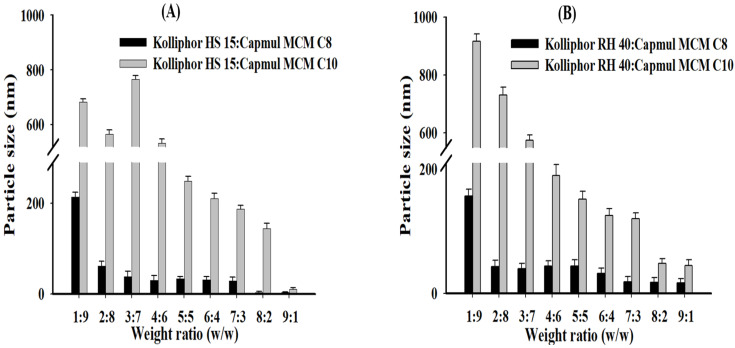
Particle size of vehicle dispersion in water according to the weight ratios between surfactant and oil; (**A**) Kolliphor HS 15:Capmul MCM C8 and Kolliphor HS 15:Capmul MCM C10; (**B**) Kolliphor RH 40:Capmul MCM C8 and Kolliphor RH 40:Capmul MCM C10.

Vehicles without ALH were prepared by mixing the selected surfactants (Kolliphor RH 40 and Kolliphor HS 15) and oils (Capmul MCM C8 and Capmul MCM C10) at various weight ratios (surfactant:oil = 1:9–9:1). These samples were then used to determine particle size or dispersibility in water. Particle size tended to decrease with an increase of the ratio of surfactant to oil in most vehicle compositions (see Figure 3). The average particle size of the vehicle containing Capmul MCM C8 was much smaller than the Capmul MCM C10. Capmul MCM C8 provided very small and homogeneous dispersed particles. The shorter length of the carbon chain (C8 vs. C10) in the Capmul MCM C8 implemented smaller particle size (<100 nm) compared to the Capmul MCM C10 [29]. As a result, proper weight ratios of surfactant to oil were at 7:3–9:1 in the tested vehicles, which also exhibited nanoemulsion of the particle size <200 nm and no phase separation.

In previous studies by Matsaridou et al. (2012), it was reported that the emulsion stability was affected by the HLB of the surfactant and the oil/surfactant ratio. In addition, it was reported that the HLB range of 10–15 contributes to the formation of stable emulsions with finer droplet diameters [30]. In a previous study using Kollipor RH 40 and Kolliphor HS 15, the droplet sizes were 100–400 nm, and in this study, using the same surfactant, the droplet sizes were 200 nm or less [30]. Therefore, the surfactant HLB values used in this study were 14.3 (Kolliphor RH 40) and 16.3 (Kolliphor HS 15), and the vehicle component ratios showed small droplet sizes, indicating that stable emulsion formation is possible.

### 3.4. ALH-Loaded SNEDDS Solubility

ALH-loaded SNEDDS and Su-SNEDDS were prepared with the selected vehicle formulations and weight ratios as shown in Table 2. The solubilizing methods of ALH were agitation and ultrasonication.

The solubility and dispersion particle size are summarized in Table 3. The solubility of ALH in all SNEDDS prepared by agitation alone increased at the highest surfactant:oil ratio of 9:1 due to the higher solubility of the surfactant. All the SNEDDS gave good nanoemulsion dispersion with the particle size of <200 nm. As compared to the oils, the solubility was similar, but the particle size was smaller with Capmul MCM C8 than Capmul MCM C10, as in the vehicle characterization. The solubility with agitation was low with the solubility range of 0.23–0.5%. In the preparation by applying agitation and ultrasonication, some SNEDDSs (F4, F5, F7, F8, F10, and F11) showed a higher solubility as ultrasonication was efficient for making a more saturated solution of ALH with the exposure to the high sonication energy [31]. In the case of the SNEDDS (F10), the solubility of ALH increased up to 2-fold or more than agitation alone (0.23 ± 0.05% of agitation vs. 0.52 ± 0.01% of ultrasonication). The higher solubility from ultrasonication resulted in a slight increase in particle size, but all dispersion particles were in acceptable, good nanoemulsion ranges. However, SNEDDS were confirmed to have a maximum solubility of only 0.57%. This solubility was too low to afford the commercially available dose strength of 150 mg/capsule when manufactured with SNEDDS, and an additional increase in drug loading was required to improve the convenience of intake compared to conventional capsules.

The Su-SNEDDS was prepared to highly increase the drug loading%. Referring to the previous results, formulations with a high surfactant ratio of 9:1 (F3, F6, F9, F12) were selected due to the high solubility and small particle size of dispersion. The Su-SNEDDS was prepared by using agitation and ultrasonication method for reducing the particle size of the suspended ALH. The ALH loading in the Su-SNEDDS was increased to 1%, 2%, 5%, and 10%. The loading% of ALH (10%) in the Su-SNEDDS was increased to 20-fold higher than that of SNEDDS (0.5%).

### 3.5. Dissolution

**Figure 4:**
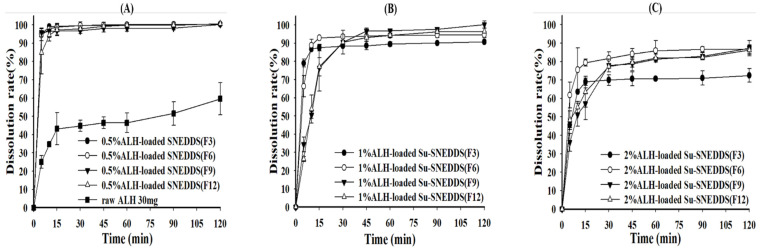
Dissolution profiles of raw ALH (**A**), 0.5% ALH-loaded SNEDDS (**A**) and Su-SNEDDS (**B**), 1% ALH-loaded; ((**C**) 2% ALH-loaded) prepared from F3, F6, F9, F12 in pH 1.2 buffer. Vehicle composition F3, F6, F9, and F12 are from Table 2.

The dissolution test was performed for 0.5% ALH-loaded SNEDDS and 1% and 2% ALH-loaded Su-SNEDDS in the pH 1.2 buffer and 1% SLS pH 1.2 buffer considering the poor water solubility of ALH [32]. First, the dissolution curves of ALH-loaded SNEDDS and Su-SNEDDS (F3, F6, F9, F12) are shown in Figure 4. All 0.5% ALH-loaded SNEDDS showed a complete dissolution rate of >95% in the pH 1.2 buffer at 15 min, whereas raw ALH showed a poor dissolution rate of 43.16 ± 8.80% at that time point (Figure 4A). This indicates that the SNEDDS enhanced the dissolution rate with forming stable dispersion of nanoemulsion without any ALH precipitation. In the dissolution of 1% and 2% ALH-loaded Su-SNEDDS, the dissolution rate in the steady state was reduced to around 90% or less with the increase of suspended ALH% (see Figure 4B,C). However, the dissolution rate for 1% and 2% ALH-loaded Su-SNEDDS was higher than the expected based on the solubilized portion of ALH (solubilization portion, 50% for 1% ALH-loaded Su-SNEDDS, and 25% for 2% ALH-loaded Su-SNEDDS, respectively). For example, 1% and 2% ALH-loaded Su-SNEDDS (F6) resulted in 92.93 ± 0.57% and 79.34 ± 1.88%, respectively, at 15 min. These data suggest that a fair amount of the suspended ALH particles were instantly dissolved in the pH 1.2 buffer with the dispersion from spontaneous nanoemulsion forming [33]. Among the tested formulations, F6 (Kolliphor HS 15:Capmul MCM C10 = 9:1, w:w) showed the highest dissolution rate compared to the other formulations.

**Figure 5:**
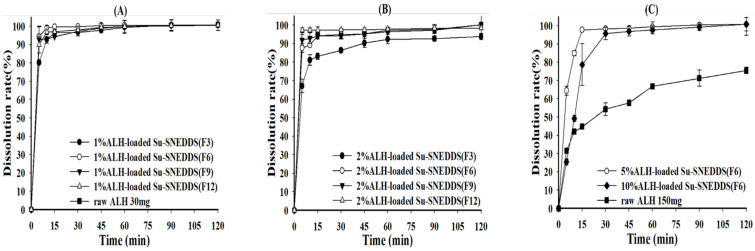
Dissolution profiles of raw ALH ((**A**) 30 mg ALH; (**C**) 150 mg ALH) and ALH-loaded Su-SNEDDS ((**A**) 1% ALH-loaded; (**B**) 2% ALH-loaded; (**C**) 5% or 10% ALH-loaded) prepared from F3, F6, F9, and F12 in 1% SLS pH 1.2 buffer. Vehicle composition F3, F6, F9, and F12 are from Table 2.

As the final dissolution rate did not reach 100% in the pH 1.2 buffer for the Su-SNEDDS, a further dissolution test was performed using the 1% SLS pH 1.2 buffer as a medium (Figure 5). The 1% and 2% ALH-loaded Su-SNEDDS showed a high dissolution rate compared to raw materials, and the Su-SNEDDS (F6 and F9) reached more than 95% at 15 min, which provided the complete dissolution rate (Figure 5A,B). F6 was the optimized formulation in terms of high dissolution rate between the tested formulations. The SLS presented in the medium promoted the dissolution with forming micelle instantly when the suspended ALH in the Su-SNEDDS was dispersed. Moreover, the dissolution test for the 5% and 10% ALH-loaded Su-SNEDDS (F6), which showed the highest dissolution rate in the lower ALH-loading%, was performed (Figure 5C). When compared to the 1 and 2% ALH-loaded Su-SNEDDS, the initial dissolution rate of the 5% and 10% ALH-loaded Su-SNEDDS (F6) was delayed as the ALH loading increased to 5% and 10%. However, the dissolution rate of the both the Su-SNEDDS in the steady state reached more than 95% within 30 min. This result showed that the optimized ALH-loaded Su-SNEDDS contains 150 mg of ALH, which is equivalent to a commercial capsule dose, and it is possible to design a dosage form with enhanced solubility, dissolution, and convenience of intake [34].

### 3.6. Microscopic Observation Evaluation

**Figure 6:**
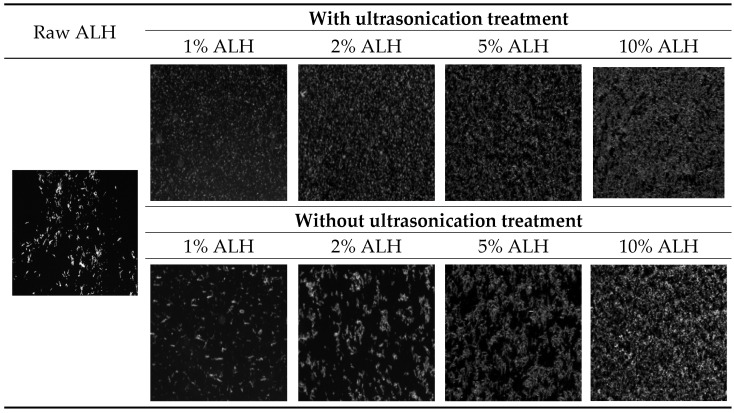
Optical microscope observation of ALH particles from raw ALH, ALH-loaded Su-SNEDDS (F6) with or without ultrasonication treatment.

For the explanation of the enhanced dissolution of the Su-SNEDDS, microscopic observation of ALH particles in a representative Su-SNEDDS (F6) and their dispersion in the dissolution medium was performed (see Figure 6). The ALH particles in the Su-SNEDDS were very small and, probably due to the ultrasonication crushing, were evenly spread without any agglomeration. However, the ALH particles without any ultrasonication were of a larger size and agglomerated together. The raw ALH particles were in the shape of a long rod. The particles were agglomerated more as the ALH% of the suspension increased. Therefore, the presence of ultrasonication method made the Su-SNEDDS (F6) a more micronized and homogeneous suspension system as well as enhanced the dissolution with rapid emulsifying and reduced precipitation [35].

**Figure 7:**
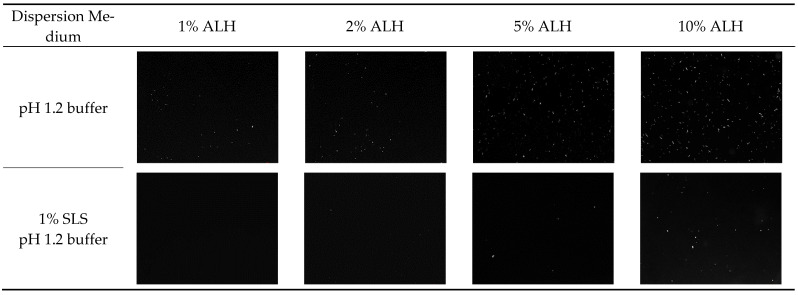
Optical microscope observation of ALH particles from the dispersion of ALH-loaded Su-SNEDDS (F6) in pH 1.2 buffer and 1% SLS pH 1.2 buffer.

Using ALH-loaded Su-SNEDDS (F6), which showed the highest dissolution rate, the ALH particles were observed in the dispersion of the pH 1.2 buffer and the 1% SLS pH 1.2 buffer by the 100-fold dilution (see Figure 7). The reason for the dilution was to observe the particles in a similar condition to the dissolution test. The observed particles were in the micronized rod shape, and the number of particles increased with the higher ALH%. Most of the suspended small particles observed in Figure 6 (1%, 2%, 5%, 10% ALH with ultrasonication) would be instantly dissolved in the dispersion medium, and the relatively larger particles remained and were observed since these particles takes time to get completely dissolved. The number of ALH particles was less in the 1% SLS pH 1.2 buffer as compared to the pH 1.2 buffer. Thus, this observation explained the decrease in the maximum dissolution rate according to the ALH% and the increase in the dissolution rate by SLS. In conclusion, the ALH-loaded Su-SNEDDS (F6) was previously reported to be a promising solubilization technology to enhance the dissolution of ALH in a novel working way of suspension [36].

### 3.7. Scanning Electron Microscope (SEM)

**Figure 8:**
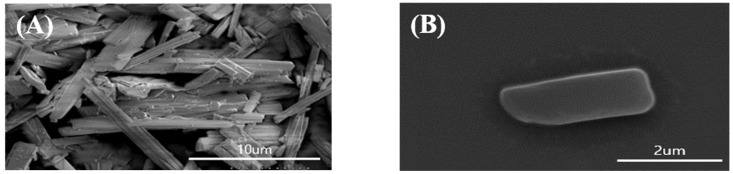
The SEM images of ALH particles from raw ALH (**A**) and dispersion of 2% ALH-loaded Su-SNEDDS (F6) (**B**) in pH 1.2 buffer.

SEM images of raw ALH and suspended ALH particles in 2% ALH-loaded Su-SNEDDS (F6) which gave good dissolution profiles were obtained (see Figure 8). Raw ALH was long and rod-shaped, and the particle size was uneven. The suspended ALH particle had very small fragments of <5 μm in length due to ultrasonication. These micronized ALH particles in the Su-SNEDDS could be efficiently dispersed to the dissolution medium with the dispersing forces to form nanoemulsions, and the solubilization of ALH was promoted [37].

## 4. Conclusions

In the present study, a novel Su-SNEEDS containing poorly soluble ALH was developed and presented. Saturated, solubilized SNEDDS was prepared by the agitation and ultrasonication method, and a suspension method was selected to increase drug loading and further increase solubility. Homogenized ALH-loaded Su-SNEDDS with a high drug loading% were successfully prepared using suspension and ultrasonication. Ultrasonication reduced particle size and uniformly distributed particles. This study shows that increasing drug loading of the ALH can increase dosing convenience, solubility, and the dissolution rate. However, since it is in a suspended form, there may be a risk to physical stability during storage due to Ostwald ripening, and it is necessary to predict the shelf life through long-term stability evaluation. Su-SNEDDS (F6) is an advanced SNEDDS formulation that increases drug loading and solubility of poorly soluble drugs. Based on the results, it can be concluded that the Su-SNEDDS can act as an improved dissolution enhancement strategy from a clinical perspective.

## Figures and Tables

**Table 1:** Solubility by pH and SLS concentration. All data are expressed as the mean ± SD (*n* = 3).

Test Solution	Solubility (μg/mL)	Test Solution	Solubility (μg/mL)
pH 1.2	8.89 ± 1.87	1% SLS	194.35 ± 5.14
pH 4.0	1.02 ± 0.15	3% SLS	211.95 ± 2.31
pH 6.8	11.02 ± 0.32	5% SLS	222.34 ± 3.38
water	38.27 ± 0.19	10% SLS	754.31 ± 5.32

**Table 2:** Vehicle compositions for ALH-Loaded SNEDDS and Su-SNEDDS.

Vehicle Composition (Weight Ratio %)	F1	F2	F3	F4	F5	F6	F7	F8	F9	F10	F11	F12
Kolliphor HS 15	70	80	90	70	80	90	0	0	0	0	0	0
Kolliphor RH 40	0	0	0	0	0	0	70	80	90	70	80	90
Capmul MCM C8	30	20	10	0	0	0	30	20	10	0	0	0
Capmul MCM C10	0	0	0	30	20	10	0	0	0	30	20	10

**Table 3:** Solubility and particle size for the ALH-loaded SNEDDS prepared from agitation and ultrasonication. All data are expressed as the mean ± SD (*n* = 3).

Formulation	ALH-Loaded SNEDDS			
	Agitation		Agitation and Ultrasonication	
	Solubility (*w*/*w*%)	Particle Size (nm)	Solubility (*w*/*w*%)	Particle Size (nm)
F1	0.35 ± 0.04	63.87 ± 11.40	0.37 ± 0.02	163.42 ± 3.51
F2	0.48 ± 0.01	5.43 ± 4.79	0.57 ± 0.01	25.03 ± 6.22
F3	0.49 ± 0.06	4.33 ± 7.51	0.56 ± 0.02	14.77 ± 1.40
F4	0.27 ± 0.02	173.77 ± 7.87	0.52 ± 0.05	199.80 ± 8.12
F5	0.27 ± 0.02	158.57 ± 4.32	0.52 ± 0.02	169.03 ± 15.09
F6	0.49 ± 0.01	138.57 ± 5.61	0.57 ± 0.02	149.72 ± 11.33
F7	0.24 ± 0.05	21.13 ± 3.96	0.42 ± 0.02	32.65 ± 4.38
F8	0.43 ± 0.04	20.03 ± 0.83	0.57 ± 0.01	33.89 ± 2.41
F9	0.45 ± 0.03	14.53 ± 0.60	0.56 ± 0.01	21.45 ± 1.67
F10	0.23 ± 0.05	141.37 ± 3.46	0.52 ± 0.01	153.67 ± 2.80
F11	0.29 ± 0.03	23.43 ± 3.17	0.53 ± 0.02	42.37 ± 4.10
F12	0.30 ± 0.07	13.53 ± 0.55	0.52 ± 0.01	18.93 ± 2.25
